# Subcellular Targeting of Theranostic Radionuclides

**DOI:** 10.3389/fphar.2018.00996

**Published:** 2018-09-04

**Authors:** Bas M. Bavelaar, Boon Q. Lee, Martin R. Gill, Nadia Falzone, Katherine A. Vallis

**Affiliations:** CR-UK/MRC Oxford Institute for Radiation Oncology, Department of Oncology, University of Oxford, Oxford, United Kingdom

**Keywords:** subcellular targeting, radioimmunotherapy, targeted radionuclide therapy, radiopharmaceuticals, cancer, dosimetry

## Abstract

The last decade has seen rapid growth in the use of theranostic radionuclides for the treatment and imaging of a wide range of cancers. Radionuclide therapy and imaging rely on a radiolabeled vector to specifically target cancer cells. Radionuclides that emit β particles have thus far dominated the field of targeted radionuclide therapy (TRT), mainly because the longer range (μm–mm track length) of these particles offsets the heterogeneous expression of the molecular target. Shorter range (nm–μm track length) α- and Auger electron (AE)-emitting radionuclides on the other hand provide high ionization densities at the site of decay which could overcome much of the toxicity associated with β-emitters. Given that there is a growing body of evidence that other sensitive sites besides the DNA, such as the cell membrane and mitochondria, could be critical targets in TRT, improved techniques in detecting the subcellular distribution of these radionuclides are necessary, especially since many β-emitting radionuclides also emit AE. The successful development of TRT agents capable of homing to targets with subcellular precision demands the parallel development of quantitative assays for evaluation of spatial distribution of radionuclides in the nm–μm range. In this review, the status of research directed at subcellular targeting of radionuclide theranostics and the methods for imaging and quantification of radionuclide localization at the nanoscale are described.

## Introduction

The capacity of a pharmacon to selectively find its biological target is an important determinant of its usefulness in clinical medicine. Many pharmacologically active substances have intracellular molecular targets that reside in organelles (D’Souza and Weissig, 2009). Carriers that selectively target these subcellular structures have been investigated extensively, and include micro- or nanoparticulate drug carriers, liposomal formulations, macromolecular drug conjugates, and chemically modified proteins (Rajendran et al., 2010). One field where subcellular targeting has been relatively unexplored, but has the potential to make a profound impact, is targeted radionuclide therapy (TRT).

Targeted radionuclide therapy is a treatment modality that encompasses the use of radionuclide-conjugated cancer-specific vectors, such as small molecules, peptides, antibodies, and nanoparticles, to selectively deliver radiation to the tumor. TRT compounds are often designed as theranostic agents; so that, when coupled to suitable radionuclides, they can be used for positron emission tomography (PET) or single-photon emission computed tomography (SPECT). A large body of evidence, accumulated over several decades, has established TRT as an effective anticancer strategy (Jackson et al., 2013; Pouget et al., 2015; Aghevlian et al., 2017). Prominent examples include treatments for lymphoma [yttrium-90 (^90^Y)-ibritumomab tiuxetan], neuroblastoma [iodine-131 (^131^I)-MIBG], and prostate cancer [radium-223 dichloride (^223^RaCl_2_)] (Larson et al., 2015; Gill et al., 2017). The success of the NETTER-1 trial with lutetium-177 (^177^Lu)-DOTATATE in patients with midgut neuroendocrine tumors (Strosberg et al., 2017), and the promising results of ^177^Lu-prostate specific-membrane antigen (^177^Lu-PSMA) ligand treatments in patients with prostate cancer (von Eyben et al., 2018) have given this field further positive momentum. However, challenges remain, including heterogeneous expression of molecular targets relevant to TRT, sub-optimal tumor delivery or penetration, and radioresistance. The latter characteristic means that for many solid tumors a five- to 10-fold higher radiation absorbed dose must be achieved for tumor eradication compared to hematological malignancies (Pouget et al., 2011). Several strategies have been tested to increase the anticancer efficacy of TRT. One option is to design radiopharmaceuticals that specifically target radiosensitive organelles to increase the probability of cell kill (Aghevlian et al., 2017). Typically, radionuclides that emit short-range Auger electrons (AEs) are used in this context due to their highly localized dose-deposition. It is notable that the decay of several widely-used β-emitting therapeutic radionuclides includes a substantial AE contribution, thus generating both local and distant radiotoxic effects due to AE and β-electrons, respectively (Falzone et al., 2015). Subcellular targeting of these radionuclides may be advantageous since the AE cause local damage to the targeted compartment. Even for high energy, short range α-emitters, the effects of subcellular localization may influence the cytotoxicity of short-range daughter products. Therefore, a better understanding of the subcellular distribution of radionuclides may lead to optimization of TRT. Over the past decade, several strategies for subcellular delivery have been tested, including nuclear, mitochondrial, cell membrane, and lysosomal delivery. This review is focused on the relationship between the track path-length of radionuclide emissions, subcellular targeting, and radiation-induced cell kill. To understand the importance of subcellular targeting in TRT, basic radiobiological concepts will be reviewed. The methods used to investigate the subcellular distribution of radionuclides are considered and the results of pre-clinical and clinical studies aimed at exploring organelle-directed TRT are evaluated.

## Radiobiology of Targeted Radionuclide Therapy

### Radionuclide Properties

Radionuclides are unstable atomic nuclei that release energy by emission of particulate radiation in the form of α-particles, β-particles, or AE, and by electromagnetic radiation in the form of X- or γ-rays. Their action is described by their linear energy transfer (LET), which is the amount of energy that an ionizing particle deposits in matter per unit distance. Radionuclides are used extensively for diagnostic and therapeutic purposes in cancer treatment. β electron-emitting radionuclides, also known as β-emitters, have historically been the most commonly used class of radionuclides in the therapeutic setting. β-emitters release electrons of mean energy ranging from about 0.2–1.0 MeV, resulting in a long track path-length with a continuous-slowing-down-approximation (CSDA) range in water of up to 12 mm. As a consequence, the LET of β-emitters is low (<1 keV/μm; Kassis, 2004). With the clinical development of ^223^RaCl_2_ over the last decade, α-emitters are now being studied intensively for various applications. α-emitters decay by releasing helium nuclei, known as α-particles, with energy ranging from 5 to 9 MeV over an intermediate track path-length in water (50–100 μm), resulting in high LET to exposed cells (50–230 keV/μm; Pouget et al., 2015). AEs, which are of particular interest in the context of subcellular targeting, are ejected from electron shells following a process called internal atomic ionization. This process is a result of nuclear decay modes that interact strongly with atomic shells, such as electron capture or internal conversion. Most AEs have a low energy (<5 keV) and very limited tissue penetration depth (< 1 μm), leading to a high LET (4–26 keV/μm; Lee et al., 2015). It is important to note that most radionuclides emit multiple types of radiation. For example, ^177^Lu, which is used in the treatment of somatostatin receptor positive tumors (^177^Lu-DOTATATE) and in the prostate cancer-targeting agent, ^177^Lu-PSMA, decays by emission of both β-electrons and AE (Hindié et al., 2016). Furthermore, photons emitted during the decay can be detected by SPECT, which makes it a suitable radionuclide for combined therapy and diagnosis (Uribe et al., 2017). **Table 1** summarizes the decay properties of several radionuclides that have been used or considered for diagnostics and therapy in the clinic (Eckerman and Endo, 2007; Falzone et al., 2015).

**Table 1:** Decay properties of several radionuclides used in diagnostics and therapy.

Per decay	^67^Cu_(β)_	^67^Ga_(A)_	^99m^Tc_(A)_	^111^In_(A)_	^123^I^∗^_(A)_	^153^Sm_(β)_	^161^Tb_(β)_	^177^Lu_(β)_	^211^At_(α)_
Half- life (days); Decay mode	2.58; β^-^	3.26; EC^f^	0.25; IT^g^ β^-^	2.80; EC	0.55; EC	1.93; β^-^	6.89; β^-^	6.65; β^-^	0.30; EC α
Yield of AE^a^ and CK^b^ e^-^	0.56	4.96	4.41	7.43	13.7	6.58	11.0	1.12	6.53
Yield of IE^c^ e^-^	0.15	0.34	1.10	0.16	0.16	0.81	1.42	0.15	3.85E-04
Yield of X-rays	0.78	6.87	5.58	9.50	15.8	8.30	13.0	1.37	7.73
Yield of γ rays	0.73	0.87	0.89	1.85	0.86	0.37	0.53	0.18	1.38E-02
Yield of β^+^ or β^-^	1.00	–	3.70E-05	–	–	1.00	1.00	1.00	–
Yield of α	–	–	–	–	–	–	–	–	1.00
Yield of α recoils	–	–	–	–	–	–	–	–	1.00
Total γ- and X-ray energy (keV/nt)	115	160	127	386	173	64.3	36.5	35.1	44.8
Total β^+^ or β^-^ energy (keV/nt)	136	–	4.20E-03	–	–	224	154	133	–
Total IE e^-^ energy (keV/nt)	13.7	29.7	15.2	27.9	21.0	40.3	39.3	13.5	0.27
Total AE and CK energy (keV/nt)	0.75	6.64	0.94	6.88	7.23	6.02	8.94	1.13	5.86
Total α energy (keV/nt)	–	–	–	–	–	–	–	–	6.78E+03
Total α recoil energy (keV/nt)	–	–	–	–	–	–	–	–	131
Total energy released (keV/nt)	266	196	1.43	441	201	334	239	183	6.96E+03
(p/e)^d^ ratio	0.76	4.50	7.90	11.1	6.10	0.24	0.18	0.24	6.48E-03


*Yield is the number of radiative species released per decay (/nt). Source: Falzone et al. (2015). Adapted and reproduced with permission from the Journal of Nuclear Medicine. Copyright: Society of Nuclear Medicine and Molecular Imaging.*

*A: SPECT; B: PET; ^∗123^I decays to ^123^Te and ^123m^Te, with half-lives too long to play a role in TRT. ˆ^211^At decays to ^211^Po (T_1/2_ = 0.516 s) and ^207^Bi (T_1/2_ = 31.55 y), radiation from the latter is excluded due to its long half-life.*

*^a^Auger electron; ^b^Coster–Kronig electron; ^c^internal conversion electron; ^d^ratio of penetrating to non-penetrating ionizing radiation; ^e^specific activity; ^f^electron capture; ^g^isomeric transition.*

**FIGURE 1:**
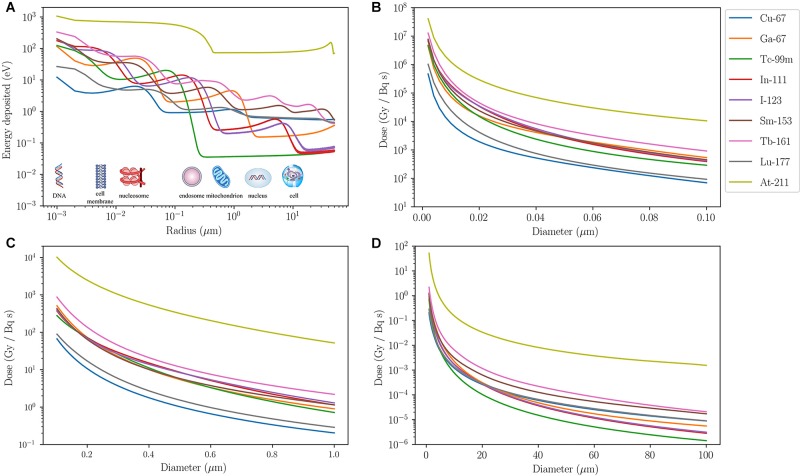
Energy and dose-deposition of various radionuclides. Energy and dose-deposition profiles for various radionuclides in spherical water volumes up to 100 μm in diameter. Calculations for electrons are based on a Monte Carlo method described by Falzone et al. (2017). Energy deposition by α-particles and recoiling daughter nuclei is derived from the NIST (Berger et al., 2005) and SRIM (Ziegler et al., 2010) stopping power data, respectively, and a straight projectile is assumed. **(A)** Energy deposited (eV) in 1-nm-thick spherical shells as a function of radius (μm) from a point source. The energy-deposition profiles exhibit sinusoidal behavior, apart from the α-emitter ^211^At, due to AE emitted from different atomic shells being stopped at different distances. β-emitters (^67^Cu, ^153^Sm, ^161^Tb, and ^177^Lu) eventually overtake the AE-emitters after 10 μm from the point source. ^211^At deposits at least an order of magnitude higher energy than other radionuclides for the entire range considered. Its energy-deposition profile is fairly constant from 0.3 μm, where all recoiling daughters are stopped, up to about 30 μm where some α-particles emitted from ^211^At start to slow down and eventually come to a halt around 50 μm. **(B–D)** Absorbed dose in spherical volumes per cumulated activity (Gy/Bq/s) as a function of diameter (μm) with a point source at the center. AE-emitters deposit significantly more dose than β-emitters that have a small AE contribution (^67^Cu and ^177^Lu) in organelles with diameter less than 1 μm. For volumes bigger than a cell, β-emitters are more suitable in delivering the desired dose over the entire region. This highlights the need for AE-emitters to be targeted to radiosensitive subcellular organelles for the anticipated therapeutic efficacy. Although ^161^Tb is a β-emitter, its decay involves a significant contribution of AE so subcellular targeting using this radionuclide would enhance its radiobiological effect. The dose-deposition profile of ^211^At shows that it delivers a significantly higher physical dose to a spherical volume of diameter less than 100 μm than the other radionuclides considered.

To illustrate how the different ranges of these particles translate to successful subcellular targeting, the associated absorbed dose per cumulated activity for different radionuclides are compared in **Figure 1**. Most of the energy associated with AE is deposited over a distance < 1 μm from the point of decay. Examples include gallium-67 (^67^Ga), technetium-99m (^99m^Tc), indium-111 (^111^In), and iodine-123 (^123^I) (**Figure 1A**). A sinusoidal energy-deposition profile is seen for all AE-emitting radionuclides in the first 10 μm from the point of decay. A similar profile, albeit less pronounced, is also present for β-emitters, i.e., copper-67 (^67^Cu), samarium-153 (^153^Sm), terbium-161 (^161^Tb), and ^177^Lu, as they also emit AE. When considering the absorbed dose received by targeted organelles within a cell, it can be seen from **Figures 1B–D** that AE-emitters are significantly more effective than β-emitters ^67^Cu and ^177^Lu for organelles such as the mitochondria. It is noteworthy that the β-emitting radionuclide ^161^Tb deposits a higher dose over the distance considered than the other β-electron-emitting radionuclides, which is partly due to release of AE in the decay cascade. β-emitters, on the other hand, deposit a greater absorbed dose than AE-emitters when the target volume is larger than an average cell (∼20 μm in diameter). α-emitters, such as astatine-211 (^211^At), deliver the highest dose within the range considered. At dimensions larger than a standard cell diameter (20 μm), ^211^At dose is at least two orders of magnitude higher than β- and AE-emitters.

### Radiobiological Implications of Radionuclide Therapy

As a result of their low LET and long track-length, β-electrons cause sparse ionizations over tens to hundreds of cell diameters. The range of β-electrons means that a vector carrying radionuclides of this type may not need to reach each and every tumor cell to achieve the desired anti-tumor effect as non-targeted cells may be damaged through the “crossfire effect” (Pouget et al., 2015). Thus, success of this type of therapy is not critically dependent on homogeneous radionuclide distribution within a tumor. On the downside, this also means that nearby normal tissue may receive a toxic absorbed dose, thus limiting the administered amount of radioactivity and, therefore, the tumor absorbed radiation dose that can be safely achieved. Furthermore, the low-density ionizations caused by β-electrons may not be sufficient to cause cell death, especially in radioresistant cells. In contrast, α-particles travel a much shorter distance (roughly 5–10 cell diameters) and, in contrast to β-emitters, are densely ionizing and cause complex, irreparable DNA damage in the cells they traverse. An intriguing phenomenon for TRT with α-emitters is dissociation of the radionuclide from the chelating group upon decay, either due to the altered coordination chemistry of the daughter elements in the decay cascade or the high recoil energy generated upon the emission of α-particles. The recoiled daughter nuclei are often themselves α-emitters and this phenomenon may lead to toxicity or desired cytotoxic effect at the daughter decay sites as some daughter nuclei are long-lived and able to move away from the targeted cells before they decay (Dekempeneer et al., 2016; Ackerman et al., 2018). Furthermore, these recoiled daughter nuclei have a LET that is at least 10 times greater than the ejected α-particles and may contribute significantly to ionization events in the immediate vicinity of the decay site (Kozempel et al., 2018). The extreme density of ionization events caused by α-emitters and their daughter nuclides, which are short-lived and decay close to the decay site of the parent nuclide, may be exploited for therapeutic gain in TRT if directed at specific radiosensitive cellular compartments. AE cause dense ionizations, but only on a nanometer scale. The result is that the radionuclides are relatively harmless unless they are in close proximity to a targeted radiosensitive subcellular structure (Falzone et al., 2015). This characteristic can be highly advantageous in reducing the side effects of TRT as, unlike α- or β-particles, non-targeted neighboring cells remain unaffected by radiation. For the radiopharmaceutical to be effective in the targeted cell, however, precise subcellular targeting is essential for the radiopharmaceutical to achieve its optimal anti-tumor effect. As many β-emitters also emit AE, their anti-tumor effect in the targeted cells could be enhanced if precise subcellular targeting is applied to β-emitters. An example of this is ^161^Tb, a β-emitter that emits a comparable number of AE to many classical AE-emitters.

Besides targeted radiation effects, TRT can also induce non-targeted effects, which have been hypothesized to result from the production of various apoptotic factors, cytokines, and reactive oxygen species (ROS). This is known as the “bystander effect,” causing perturbations in unirradiated cells which are close to irradiated cells. The bystander response is observed for both high-LET (α-particles and AE) and low-LET (β-electrons) radiation (Boyd et al., 2006). The topic of the bystander effect has been recently reviewed (Brady et al., 2013; Pouget et al., 2015).

## Methods to Detect the Spatial Distribution of Radionuclides

To evaluate the potential efficacy of a novel radionuclide therapeutic, it is essential to obtain information about the spatial distribution and radiation dose deposition in relevant tissues and cells. One of the major advantages of TRT is the ability to visualize drug distribution and tumor targeting in patients by means of PET or SPECT. Isotopes such as ^99m^Tc, ^111^In, and ^123^I were initially used for diagnostic purposes due to the emission of γ-rays, and only later considered as potential therapeutics as a result of the AE-emitting effects (Gomes et al., 2011). Cellular targeting was mainly directed to membrane receptors, because of their abundant expression and relative accessibility. However, the majority of potential cancer cell targets reside intracellularly (Cornelissen, 2014). Specific subcellular targeting of theranostic probes may therefore not only have an impact on cancer cell kill, but also on imaging. Several techniques have been described to elucidate the subcellular distribution of radionuclides (Puncher and Blower, 1994; Falzone et al., 2012; Mather, 2012; Kim et al., 2017). This section reviews the different techniques and provides a summary of the main advantages and limitations (**Table 2**).

**Table 2:** Advantages and disadvantages of subcellular localization techniques for radionuclide therapy.

Methods	Radionuclides	Advantages	Disadvantages
Fractionation assay	γ-emitters	Quantitative	Disruptive
		Ease of use	No spatial information
		Required time (2–3 days)	Does not take population variation into account
EM-MAR	AE-emitters	High spatial resolution	Fixed cells
		Semi-quantitative	High rate and ease of artifact production
			Non-linear signal due to silver bromide crystal saturation
			Required time (1.5–3 weeks)
			Specialist handling required
PAR	AE-emitters	High spatial resolution (∼10 nm)	Fixed cells
		Semi-quantitative	Lithography process can lead to over-development
NanoSIMS	All isotopes	High spatial resolution	Fixed cells
		Quantitative	Sample preparation
		Suitable for stable and radioisotopes	Cost
			Specialist handling required
Radioluminescence	β- and positron-emitters	Potential of live cell imaging	Low throughput (∼100 cells/acquisition)
		Moderate spatial resolution	Long acquisition times (15–30 min)
		Highly sensitive (<1 attomole)	
		Quantitative	
α-camera	α-emitters	Quantitative	Low resolution (∼35 μm)
			Requires collimation
Timepix	α-, β-, γ-, and muon-emitters	Quantitative	Low resolution (∼77 μm)
		Suitable for a variety of radionuclides	


### Fractionation Assays

One of the most commonly employed methods to determine the localization of radionuclides on a subcellular level is the use of fractionation protocols for isolation of subcellular components. A gamma-counter is then used to detect and quantify the amount of radioactivity associated with each fraction. Cells may be disrupted by osmotic shock, ultrasonic vibration, lyzed in a blender, or extruded through a fine needle. These procedures are detrimental to cell membrane integrity, including the endoplasmic reticulum (ER) and plasma membrane, but leave important organelles such as the nucleus, mitochondria, peroxisomes, and lysosomes largely intact. Organelles each have a distinctive size, charge, and density, and the homogenate can therefore be separated by centrifugation steps in different buffers (Alberts et al., 2002). When these fractions are separated, and the radioactive content is measured using a γ-counter, it is possible to obtain a highly quantitative measure of the relative uptake of the radionuclide in each type of organelle. Several researchers have reported the use of this approach, including Chen et al. (2006), Zereshkian et al. (2014), and Ngo Ndjock Mbong et al. (2015). This technique can be used for all γ-emitting radionuclides, making it a highly useful technique in TRT research. Although this technique is excellent for describing the average intracellular distribution of radioactivity for cell populations, detailed spatial information is lost. Furthermore, fractionation and gamma-counting provide a population average distribution and do not take into account the large variation of uptake between individual cells.

### Microautoradiography

Microautoradiography is a technique that involves the use of X-ray films, beta imaging systems, phosphor imaging plates, or a photo-nuclear emulsion to detect radiolabeled molecules. It can be used to visualize and quantitatively resolve compounds in tissue, cells, and subcellular organelles (Solon, 2015). Two techniques are of particular relevance to the study of the localization of radiolabeled drugs to subcellular structures: electron microscopy – microautoradiography (EM-MAR) (Paul et al., 1970) and photoresist autoradiography (PAR; Falzone et al., 2011).

#### Electron Microscopy – Microautoradiography

**FIGURE 2:**
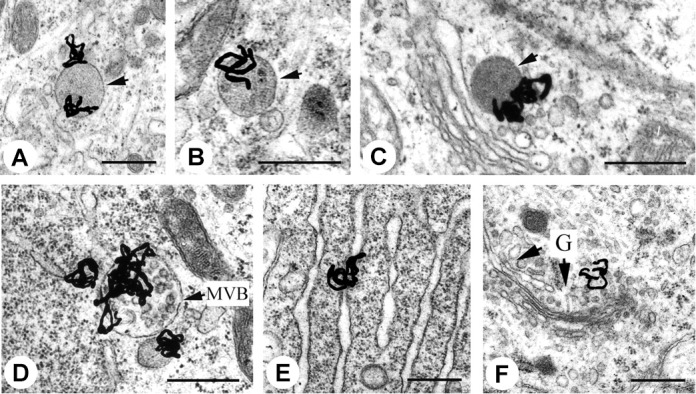
Electron microscopy – microautoradiography of radionuclides in subcellular compartments. EM-MAR images of hypoglossal motoneurons treated with ^125^I-labeled retrogradely transported trophic factors. The location of the radionuclide is revealed by the formation of silver grains. **(A)** ^125^I-labeled glial cell line-derived neurotrophic factor (GDNF) in a light endosome (arrow). **(B)** ^125^I-labeled brain-derived neurotrophic factor (BDNF) in a dense endosome (arrow). **(C)** ^125^I-labeled CT-1 in a lysosome (arrow). **(D)** ^125^I-labeled GDNF in a heavily labeled multivesicular body (MVB). **(E)** ^125^I-labeled GDNF in the endoplasmic reticulum. **(F)** ^125^I-labeled GDNF in the Golgi apparatus with Golgi (G)-associated vesicles (arrows). Scale bars represent 500 nm. Source: Rind et al. (2005). Reproduced with permission from the Journal of Neuroscience. Copyright: Society of Neuroscience.

EM-MAR is a technique that was reported soon after the development of the electron microscope by Liquier-Milward (1956), enabling visualization of the subcellular distribution of short track-length particulate radiation, such as AE. EM-MAR involves the exposure of cultured cells or excised tissues to radiolabeled compounds followed by preparation of the material for transmission EM. After mounting sections on copper grids, a fine-grain silver bromide photographic emulsion is applied in a dark room. The silver bromide crystals in the emulsion may be reduced by the radiation, and after exposure for a suitable length of time, the grids are developed photographically, resulting in transformation of reduced crystals into small black grains. The position of these grains corresponds with the localization of the radiolabeled drugs within the original sample and can give a quantitative image when measured with an EM. The high spatial resolution of the microscope provides an excellent means of imaging radionuclides in subcellular compartments (Solon, 2015). Rind et al. (2005) used this technique to measure the fate of retrograde-transport of ^125^I-labeled trophic factors in neonatal rat hypoglossal motoneurons, and were able to visualize individual grains in endosomes, lysosomes and the Golgi apparatus (**Figure 2**). Grain formation occurs when the emulsion comes into contact with AE, and can therefore be used with AE-emitting radionuclides, such as ^111^In, ^125^I, and ^67^Ga (Puncher and Blower, 1994). Despite the advantages, the technique is used only rarely, which can be attributed mainly to the long processing time (up to several weeks), the appearance of silver grain artifacts that complicate interpretation of results, and the difficult and highly specialized techniques needed to develop the TEM grids (Solon, 2015).

#### Photoresist Autoradiography

**FIGURE 3:**
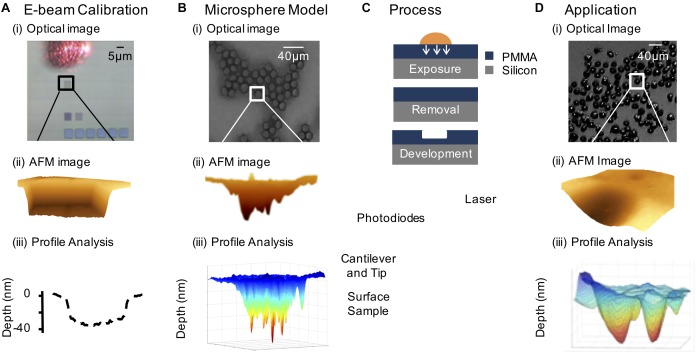
The photoresist autoradiography method. **(A)** Electron beam calibration: **(i)** 5×5 μm^2^ patterns of varying fluence incident on the PMMA substrate (the laser reflecting off the AFM probe is shown). (ii) AFM image of 5 μm × 5 μm electron beam feature. (iii) Line scan relating depth to electron fluence. **(B)** Model system consisting of ^111^In-DTPA radiolabeled microspheres: **(i)** optical image showing the close packing of the microspheres on the PMMA surface, **(ii)** AFM contour through image of a radiolabeled microsphere pattern, and **(iii)** 3-D generated profile of the AFM feature. **(C)** Resist exposed to radionuclide treated cells and isolated cell nuclei, followed by removal of biological material and chemical development of the resist and AFM analysis of the pattern. **(D)** Demonstration of PAR with ^111^In-DTPA-hEGF treated cells: **(i)** optical image of radionuclide treated SQ20B (head and neck squamous carcinoma) cells, (ii) AFM image of an ^111^In-DTPA-hEGF treated cell pattern, and (iii) 3-D generated plot of an AFM image of a cell nucleus relating local pattern depth to local fluence based on electron beam calibration.

Recently we have developed an autoradiographic technique, PAR (Falzone et al., 2011, 2012; Royle et al., 2015, 2016), based on photoresist lithography that has its origin in integrated electronics (**Figure 3**; del Campo and Arzt, 2008). This type of lithography is based on the exposure of a photosensitive polymer to AE-emitting radionuclides - vector conjugates, such as ^111^In-hEGF, which results in the pattern of distribution of the radionuclide being etched onto a polymer film (Royle et al., 2015). An atomic force microscope (AFM) is used to read the 3D-pattern in the resist, which can be used to determine the radiation dose and localization on a single-cell or subcellular level. The spatial resolution is higher than micro-autoradiography, providing an opportunity to quantify radionuclide distribution on a nanometer scale. This detailed spatial information allows a nanoscale radiation absorbed dose map to be generated. A typical experiment involves the exposure of cancer cells to a pharmacon radiolabeled using an AE-emitting isotope. Post-exposure, the cells are washed and air dried. An inverted photoresist is placed directly on top of the cells and incubated for a time roughly equivalent to four half-lives of the specific radionuclide under investigation, allowing the emitted AE to produce spatially-resolved images in poly(methyl methacrylate) (PMMA). The images are converted to a pattern of pits after chemical development, which can be measured following acquisition of AFM images.

### Nanoscale Secondary Ion Mass Spectrometry

Nanoscale secondary ion mass spectrometry (NanoSIMS) is a recent development in SIMS technology that is used to image the spatial distribution of elements, such as radionuclides, in biological and non-biological samples. NanoSIMS combines the simultaneous detection of heavy and light elements with an excellent spatial resolution (50 nm; Wedlock et al., 2013). A detailed description of the technique is given by others (Jiang et al., 2016; Nuñez et al., 2017). Briefly, in this technique, a high-energy ion beam (Cs^+^) is directed across the cell sample surface, causing atom sputtering from the topmost monolayers and resulting in the generation of negative secondary ions. These ions are mass sorted and used to produce a map of the sample surface, which shows the distribution of selected ion species. The images are produced in parallel from the same sputtered volume, allowing them to be in exact register with each other, which is necessary for acquiring quantitative images (Lechene et al., 2006). Quantitative mass images contain a number of counts at each pixel for each selected atomic mass, which is directly proportional to the sample abundance at the specific subcellular location. The high mass resolution facilitates the simultaneous detection of the intracellular distribution of multiple isotopes of the same element, which allows derivation of meaningful isotope ratios. In the case of radionuclides, a ratio higher than the natural sample abundance, which is in almost all cases very low, indicates the subcellular location and the relative excess. The high stability of primary beam, mass spectrometer, detectors, and ion optics results in precise measurements of the sample. The ability to detect stable and radioactive isotopes alike with high resolution makes this a powerful technique for the identification of the subcellular distribution of virtually every type of radionuclide (Lechene et al., 2006). The downsides of the technique are the costly, specialist, and time-consuming sample preparation, as well as its ablative nature, meaning that this technique is not suited for live cell imaging (Gao et al., 2016).

### Radioluminescence Microscopy

**FIGURE 4:**
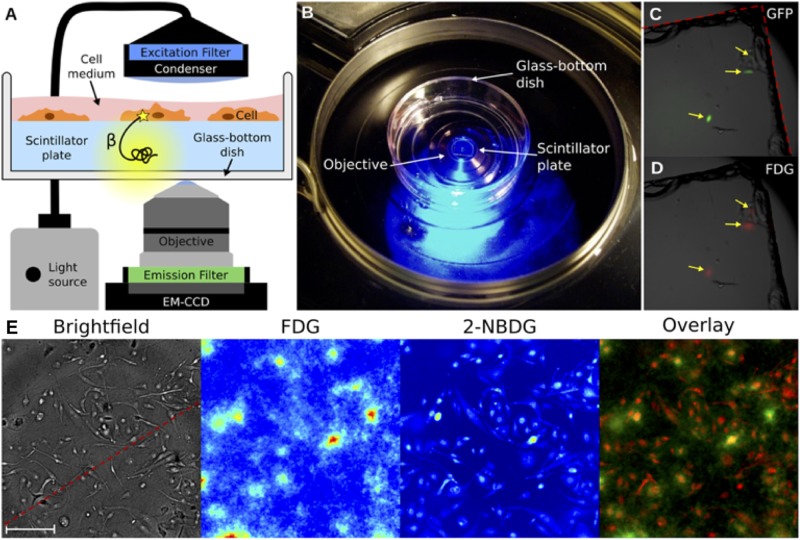
Radioluminescence microscopy. **(A)** Emission of an intracellular radionuclide can be detected as radioluminescence with a scintillator plate (yellow glow). The optical photons are captured by a high-numerical-aperture objective coupled to a deep-cooled EM-CCD camera. Concurrent fluorescence and brightfield microscopy are enabled by emission and excitation filters used in combination with a light source. **(B)** An in culture medium immersed scintillator plate in a glass-bottom dish is placed into the inverted microscope. **(C)** Three GFP-expressing HeLa cells were imaged using fluorescence microscopy. **(D)** After incubation with ^18^F-FDG the focal radioluminescence signal coincided with the fluorescent signal. **(E)** An example of radioluminescence microscopy. MDA-MB-231 cells were incubated for 1 h with ^18^F-FDG and the fluorescent 2-NBDG. Brightfield image (scale bar, 100 μm), radioluminescence (FDG), and fluorescence (2-NBDG) micrographs. The overlay shows co-localized radioluminescence (green) and fluorescence (red). Source: Pratx et al. (2012). Adapted and reproduced with permission from *PLoS One*.

Radioluminescence microscopy is a recently developed technique that can provide quantitative measurements of β-emitting radionuclide transport on the level of a single live cell (**Figure 4**; Pratx et al., 2012). Radioluminescence microscopy is based on the use of a scintillator crystal in close proximity to cells. This crystal flashes each time a β-particle or positron is emitted from the underlying cell. The individual flashes can be recorded to reveal the distribution of radiolabeled probes in single cells. The technique can be used qualitatively by using a long exposure (30–300 s) and integrating the optical signals of the many captured decays into an approximate image, or quantitatively, by obtaining many camera frames with short exposure (0.01–0.1 s). The latter requires frame-by-frame processing to extract the precise location of individual decay events, which can be digitally counted and created in a composite image (Kim et al., 2017). This technique has been pioneered by the Pratx group, who has used it in several applications, including to detect ^18^F-fluoro-deoxyglucose (FDG; Pratx et al., 2012) and ^64^Cu- and ^89^Zr-labeled rituximab (Natarajan et al., 2015). Although this technique does not provide the excellent spatial resolution of EM-MAR, it does provide the opportunity to visualize and quantify the (sub-) cellular distribution of radionuclides in live cells.

### Microdosimeters

The α-camera, first described by Bäck and Jacobsson (2010), combines autoradiography with a scintillation technique and optical registration using a charge-coupled device (CCD). It measures the activity distribution of α-particle emitters with high resolution (≤35 μm) down to a scale approaching the cellular level by virtue of the short path lengths of the α-particles. Furthermore, the pixel intensity is linearly related to the activity, thus allowing for quantitative analysis of the imaged tissue. Chouin et al. (2013) used an α-camera for the detection and measurement of an ^211^At radioimmunoconjugate that had been administered to mice with ovarian cancer micrometastases. α-camera imaging showed high uptake and retention at the tumor surface and, by measuring the activity level and the number of tumor cell clusters, it was possible to calculate dose estimates to the micrometastases.

Another microdosimeter, Timepix, takes advantage of recent developments in complementary metal–oxide–semiconductor (CMOS) technology for constructing integrated circuits (Llopart et al., 2007). Timepix consists of a silicon semiconductor layer, divided into an array of pixels, which is bumped-bonded to an electronics integrated layer. Each pixel is connected to an individual charge-sensitive preamplifier, a discriminator, a counter, and a 4-bit digital-to-analog converter (DAC) to adjust the voltage threshold (Llopart et al., 2007; Rügheimer et al., 2008). Timepix directly measures energy deposition from charged particles and photons in real time (Campbell et al., 2007). Its utility in detecting β-particles from a Carbon-14 (^14^C) sample showed that Timepix was highly sensitive with a minimum detectable activity of 0.0077 Bq and with a spatial resolution of 76.9 μm at full-width at half-maximum (FWHM; Esposito et al., 2011a,b). In another study, Timepix was used to measure α-particle emissions in tumor sections from mice treated with chemotherapy and a radiolabeled DAB4 murine monoclonal antibody [thorium-227 (^227^Th)-APOMAB] (Darwish et al., 2015). Results showed that the α-particle emissions could be visualized and quantified using the detector (Miller, 2018).

### Combining Methods

The radiographic and fractionation techniques described above can provide valuable insights into the subcellular distribution of radionuclides in their own right but additional information may be gained when they are applied in combination. For example, the spatial pattern of radionuclide deposition within cells that is derived from EM-MAR can be combined with radioactivity measurements from fractionation assays to allow the investigator to generate a precise, quantitative map of radionuclide distribution. Another option is to combine a radioactivity-detection assay with a non-radioactive assay, such as confocal microscopy or NanoSIMS (Rbah-Vidal et al., 2017). In one example, the spatial resolution of confocal microscopy was combined with quantitative fractionation assays to determine the subcellular distribution of a Cy3- or ^111^In-labeled probe targeting γH2AX (Cornelissen et al., 2011). Another example was reported by Gill et al. (2018), who evaluated the subcellular localization of ^111^In-labeled, EGF-tagged, ruthenium-loaded PLGA nanoparticles through the use of a fractionation assay. By making use of the metal to ligand charge-transfer (MLCT) “light switch” properties of the ruthenium compound, which causes a large increase in photon emission intensity when bound to DNA, it was possible to show that the majority of the nanoparticles remained in the cell cytosol while the ruthenium was also found in the nucleus; a finding confirmed by ICP-MS (inductively coupled plasma mass spectroscopy) analysis.

## Subcellular Targets for Radionuclide Therapy

### Nucleus

**FIGURE 5:**
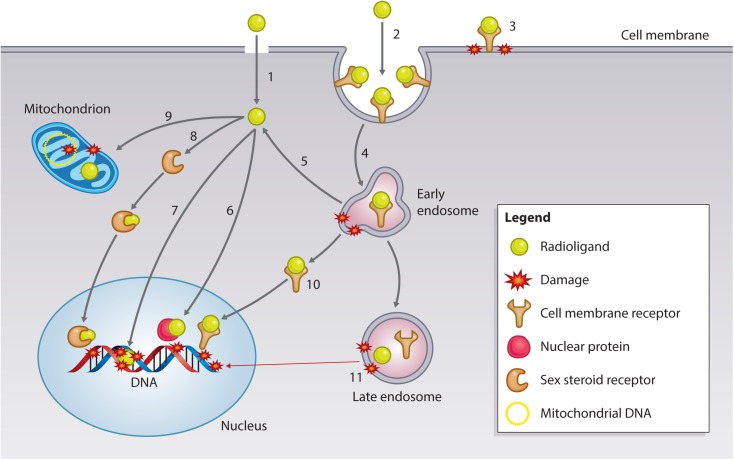
Subcellular targets of Auger electron-emitting theranostic radionuclides. Strategies to reach intracellular targets can broadly be categorized into radioligands that diffuse through the cell membrane by passive/active transport (1) or bind to cell membrane receptors. Membrane receptor-radioligand complexes can be internalized via endocytic pathways (2) or remain surface-bound (3), damaging the cell membrane via hydroxyl radical formation (see text for further explanation). Endocytosed radioligand-receptor can continue to damage endosomes (4), and certain radioligands have the potential to escape endosomal entrapment (5). Cytosolic radioligands can have various fates and targets. Approaches have utilized radioligands that can bind nuclear proteins, such as γH2AX or telomerase (6), or that can directly interact with the DNA (7). Some radioligand strategies involve targeting SSR (8), which can traffic to the nucleus of the cancer cell to exert damage. A more recently explored fate is mitochondrial targeting (9), which can lead to mitochondrial DNA damage and the generation of oxidative stress, resulting in mitochondrial-induced apoptosis. Endosomal escape can also occur for receptor-radioligand complexes, which can travel to the nucleus, as has been found with targeting of the EGFR family (10). Most complexes are unable to escape the endosome and will be sorted out of the cell via large endosomal/lysosomal vesicles. While being processed, radionuclides can continue to do damage endosomal vesicles, and irradiate genomic DNA in case of long track-path radionuclides emitters, such as ^177^Lu-/^225^Ac-PSMA or ^177^Lu-DOTATATE) (11).

Particulate ionizing radiation can damage biomolecules via one-electron oxidation reactions, the “direct effect,” or via the production of ROS, including superoxide radicals (O_2_^-^) and hydrogen peroxide (H_2_O_2_), that form the precursors of damaging hydroxyl radicals (·OH), the “indirect effect” (Pouget et al., 2015). The primary target for ionizing radiation is nuclear DNA (**Figure 5**). Particulate irradiation of the nucleus can damage the DNA indirectly via water radiolysis or directly by one-electron oxidation. These processes can result in DNA single-strand and double-strand breaks (DSBs), as well as DNA crosslinks and DNA base damage. If this damage goes unrepaired, cell death by mitotic catastrophe or apoptosis is triggered (Pouget et al., 2015). Therefore, to exploit the optimal effect of TRT, cancer-specific nuclear targeting is of interest to the research community. Several investigators have developed approaches to bring radionuclides in close proximity to cancer cell DNA. Strategies involve direct targeting of the DNA, sex steroid receptors (SSRs; androgen, estrogen), and nuclear trafficking cell surface receptors (EGFR, HER2). Furthermore, subnuclear targeting has been achieved by binding nuclear proteins (γH2AX, telomerase), and the nucleolus (Cornelissen, 2014). The following section summarizes recent advances in the area of targeting subcellular compartments (**Table 3**).

**Table 3:** Examples of radiopharmaceuticals that target subcellular compartments.

Target	Radiolabeled pharmacon	Source
Nucleus – DNA	^125^I-UDR	Kortylewicz et al., 2012
	^125^I- and ^99m^Tc-labeled acridine orange derivatives	Pereira et al., 2017
	^99m^Tc-labeled pyrene derivates	Häfliger et al., 2005; Reissig et al., 2016
	^99m^Tc-labeled doxorubicin	Imstepf et al., 2015
	^125^I-labeled daunarubicin in HER2-targeted liposomes	Fondell et al., 2011
	^99m^Tc-labeled DAPI	Kotzerke et al., 2014
	^125^I- and ^111^In-labeled TFOs	Dahmen and Kriehuber, 2012; Dahmen et al., 2016, 2017
Nucleus – nuclear proteins	^111^In-labeled anti-γH2AX antibody	Cornelissen et al., 2012, 2013
	^123^I-MST-312	Waghorn et al., 2017
Nucleus – SSRs	^123^I-labeled estrogen analogs	DeSombre et al., 1992, 2000
	^123^I-, ^125^I-, and ^131^I-labeled diethylstilbestrol	Fischer et al., 2008
	^111^In-labeled LXXLL-based peptide	Vultos et al., 2017
	^125^I-IVME2	Yasui et al., 2001
	5-^125^I-3’-*O*-(17β-succinyl-5α-androstan-3-one)-2’-deoxyuridine monophosphate	Kortylewicz et al., 2012; Han et al., 2014
Nucleus – trafficking cell surface receptors	^111^In-labeled nimotuzumab	Fasih et al., 2012
	^111^In-labeled hEGF	Cai and Chen, 2008; Vallis et al., 2014
	^67^Ga-,^111^In- and ^125^I-labeled MNT targeting EGFR, folate or melanocortin receptor	Slastnikova et al., 2012, 2017a,b; Koumarianou et al., 2014
	^125^I-labeled monoclonal antibody 425	Quang and Brady, 2004
	^111^In-trastuzumab	Costantini et al., 2007, 2008
	Methotrexate-loaded BCM conjugated to ^111^In, an NLS, and trastuzumab Fab fragments	Hoang et al., 2012
	^131^I-labeled anti-HER2 nanobody	D’Huyvetter et al., 2017
	^125^I-,^131^I-labeled anti-HER2 nanobody	Pruszynski et al., 2014
	^177^I-labeled anti-HER2 nanobody	D’Huyvetter et al., 2012
Mitochondria	^177^Lu-porphyrin-PEG nanocomplexes	Yu et al., 2018
	^99m^Tc-HMPAO (speculative)	Maucksch et al., 2016
Cell membrane	^125^I-labeled anti-CEA monoclonal antibody	Pouget et al., 2008; Santoro et al., 2009; Paillas et al., 2016
Endosomes	^177^Lu-PSMA-617	Rahbar et al., 2017
	^225^Ac-PSMA-617	Kratochwil et al., 2018
	^225^Ac-PSMA lipid vesicles	Zhu et al., 2016
	^211^At-YC-I-27	Kiess et al., 2016
	^125^I-DCIBzL	Kiess et al., 2015
	^177^Lu-DOTATATE	Strosberg et al., 2017


#### DNA-Binding Agents

Small molecules are able to bind DNA by a range of covalent and non-covalent binding modes and represent one of the most heavily studied class of anti-cancer agents. In addition, many function as fluorescent DNA dyes, and so provide a convenient means of obtaining intracellular localization information. The effect of AE-emitters on cell survival was first studied with molecules that can be incorporated in to the DNA, such as ^125^I-UDR. These studies provided valuable knowledge that illuminated the inverse relationship between AE-emitter distance to the DNA and DNA damage and cell kill (Kortylewicz et al., 2012). Since then, several small molecule DNA intercalators have been proposed as nuclear-targeting agents for AE-emitting radionuclides, such as radiolabeled derivatives of acridine orange (Pereira et al., 2017), pyrene (Häfliger et al., 2005; Reissig et al., 2016), doxorubicin (Imstepf et al., 2015), and daunorubicin (Fondell et al., 2011). Acridine orange is a cell-permeable molecule with anticancer drug and radiosensitizing properties. Pereira et al. (2017) radiolabeled several analogs with the AE-emitters ^99m^Tc and ^125^I and demonstrated that both compounds homed to the nucleus, resulting in an increased number of DSBs. The Alberto group explored the effect of ^99m^Tc-labeled pyrene-NLS (SV40 NLS peptide) conjugates on cell survival and found that these compounds exhibited a DNA damaging effect, leading to mitotic catastrophe (Haefliger et al., 2005). Reissig et al. (2016) synthesized related pyrene constructs with ^99m^Tc-labeled alkyne groups of variable length. In cell-free studies, they showed a decrease in DNA damage when the distance between the intercalating moiety was increased from 0.3 to 1.5 nm, clearly demonstrating the need for close association with DNA molecules for AE-generated DNA lesion formation. Similarly, Kotzerke et al. (2014) radiolabeled DAPI, a dual groove-binder, intercalator and commonly employed DNA dye, with ^99m^Tc. In related work, the anthracycline doxorubicin, a widely used topoisomerase II inhibitor and DNA intercalating chemotherapeutic, was conjugated to ^99m^Tc to enhance its potency and diagnostic potential (Imstepf et al., 2015). It was shown that the conjugate was readily taken up by the nucleus, caused extensive DNA damage, and exhibited a dose-dependent reduction in cell survival in several cancer cell lines. Further characterization with SPECT in nude mice revealed that ^99m^Tc-labeled doxorubicin had a similar pharmacokinetic profile as unlabeled doxorubicin, but no further *in vivo* efficacy studies were performed. Fondell et al. (2011) developed an ^125^I-labeled variant of another anthracycline, daunorubicin, and encapsulated it in HER2-targeting liposomes. This dual delivery formulation, in which HER2 targeting is used for cancer-specificity and DNA targeting to bring the AE-emitter in close proximity to its target, resulted in a high cellular uptake and significant dose-dependent reduction in cellular survival. An additional option that has been explored for targeting of cancer cell DNA is the use of triplex forming oligonucleotides (TFO), site-specific molecules that bind to the major groove of duplex DNA to form a triplex helix. Several studies by Dahmen and Kriehuber (2012) and Dahmen et al. (2016, 2017) have demonstrated that TFOs can be readily labeled with radionuclides, such as ^125^I and ^111^In, and that these conjugates can exert site- and sequence-specific DNA damage in cancer cells.

#### Nuclear Protein Targeting Agents

The nucleus not only contains DNA, but also harbors many proteins that are essential for genome expression and integrity (Cornelissen, 2014). Targeting these proteins can be exploited in TRT. One example is the development of an ^111^In-labeled antibody against γH2AX, the phosphorylated product of H2AX that forms high density foci around DNA DSBs. Cornelissen et al. (2012, 2013) demonstrated that conjugation of anti-γH2AX to the cell penetrating peptide, Tat, which is derived from the human immunodeficiency virus and contains a nuclear localizing sequence (NLS), led to increased cellular and nuclear uptake compared to non-modified antibody. Enhanced uptake of the radiolabeled Tat-modified antibody resulted in a dose-dependent reduction in clonogenic survival. Inhibition of tumor growth was seen in xenograft-bearing mice, in particular when the tumor was pre-treated with external beam radiation to induce intratumoral γH2AX expression. Furthermore, the difference in uptake between tumor and normal tissue was high enough to visualize the tumor site by SPECT, indicating the potential of this radioimmunoconjugate as a theranostic. Waghorn et al. (2017) reported the use of ^123^I-labeled small molecule inhibitors of telomerase, a ribonucleoprotein involved in telomere lengthening and cancer cell immortality. It was shown that ^123^I-MST-312, a derivate of the flavonoid epicatechin, inhibited telomerase and had a favorable uptake and nuclear distribution pattern, which resulted in a telomerase- and radioactive dose-dependent reduction in clonogenic survival after 24 h of treatment.

#### Sex Steroid Receptor Targeting Agents

Sex steroid receptors are a subclass of steroid hormone receptors that bind androgens, estrogens, and progestogens. SSRs play a pivotal role in the development and progression of malignancies, such as prostate cancer [androgen receptor (AR; Heinlein and Chang, 2004), and breast cancer (estrogen receptor (ER)/progesterone receptor (PR; Brisken, 2013; Turner et al., 2017]. Natural ligands of SSRs pass the cell membrane by simple or facilitated diffusion due to their lipophilic nature. Once the molecules are internalized, they associate with receptors in the cytosol or nucleus, following which these receptor–ligand complexes act as transcription factors for various genes. Because of their nuclear localization, SSRs have been exploited as targets for therapy with AE-emitters (Aranda and Pascual, 2001). An early experiment was conducted by DeSombre et al. (1992), who showed that ^123^I-labeled estrogen analogs result in a significant reduction in clonogenic survival of ER expressing cells. DeSombre and others subsequently evaluated various radiolabeled agents for the treatment of cancer, including ^123^I- and ^111^In-labeled analogs of estrogen and diethylstilboestrol, a non-steroid ER agonist (DeSombre et al., 2000; Yasui et al., 2001; Fischer et al., 2008; Vultos et al., 2017). Kortylewicz et al. (2012) developed an interesting hybrid molecule that exploits dual AR targeting and S-phase specific cell kill by linking 5α-dihydrotestosterone with 5-radioiodo-2′-deoxyuridine. They showed that this drug is initially trapped in the cytoplasm but associates exclusively with nuclear DNA after 24 h. A relatively low dose of radioactivity resulted in a reduction in clonogenic survival dependent on the expression of AR (Kortylewicz et al., 2012; Han et al., 2014).

#### Trafficking Cell Surface Receptor Targeting Agents

Another approach to DNA targeting is to use the nuclear trafficking properties of cell surface receptors. Although not a dominant internalization pathway, several cell surface receptors translocate to the nucleus upon ligand binding where they can act as transcription factors. Of particular interest are members of the human epidermal growth factor receptor (EGFR) family that contain NLS in the transmembrane region. Researchers, in particular the Reilly group, have exploited this concept for the nuclear targeting of AE-emitters (Aghevlian et al., 2017). The most prominently exploited target is EGFR, which is frequently overexpressed in cancer and associated with poor prognosis. Non-canonical nuclear trafficking of the receptor results in the activation of cyclin D1 and NOS, a function that is enabled by its NLS (RRRHIVRKRTLLR; Wang et al., 2010). Researchers have shown efficient cellular and nuclear uptake of ^111^In-labeled anti-EGFR immunoconjugates with and without NLS-conjugation, which resulted in effective cell kill and localization at the tumor site as visualized by SPECT in EGFR-overexpressing breast cancer MDA-MB-468 xenografts (Fasih et al., 2012). A similar effect was seen for ^111^In-labeled hEGF, that caused toxicity in cells that expressed a high number of EGFR, but not in cells with a low number of EGFR (Cai et al., 2008). Both antibody (Quang and Brady, 2004) and peptide (Vallis et al., 2014) EGFR-targeting vectors have been progressed into Phase I clinical trials, and showed the capacity to home to the tumor. This topic has been recently reviewed by Aghevlian et al. (2017). Recently, researchers have tried to further increase nuclear localization by nanoparticulate approaches, such as gold nanoparticles (Song et al., 2016) and modular nanotransporters (MNTs; Koumarianou et al., 2014). The latter strategy utilized an ingenious nanosystem, developed by Gilyazova et al. (2006), consisting of the translocation domain of diphtheria toxin (endosome escape module), an *Escherichia coli* hemoglobin-like protein (carrier module), an SV40 large T-antigen NLS peptide (nuclear import), and hEGF (ligand module), and the construct was labeled with ^67^Ga. They showed superior uptake and cytotoxicity over ^67^Ga-hEGF, which was attributed to improved nuclear retention. This MNT has been used with various other ligand modules (melanocortin receptor and folate receptor ligands) and radionuclides (^111^In, ^125^I) (Slastnikova et al., 2012, 2017a,b), and has recently been reviewed by Sobolev (2018).

A second member of the EGFR family, human EGF receptor 2 (HER2), has also been explored as a radio-theranostic target. HER2 is internalized relatively slowly and transported to the nucleus upon binding of ligands such as trastuzumab due to its NLS (KRRQQKIRKYTMRR; Giri et al., 2005). The Reilly group has extensively exploited this mechanism in combination with AE-emitter ^111^In (Costantini et al., 2007, 2008). They showed that ^111^In-trastuzumab elicited significant induction of DNA DSBs and a marked reduction of clonogenic survival. The group also exploited other vectors, such as methotrexate-loaded block copolymer micelles (BCMs) conjugated to ^111^In, an NLS (CGYGPKKKRKVGG), and trastuzumab Fab fragments. Importantly, they showed that NLS conjugation resulted in a significant increase in nuclear uptake, which led to an improved anti-proliferative effect in comparison to BCMs without NLS, highlighting the importance of subcellular targeting of AE-emitters (Hoang et al., 2012). Studies by D’Huyvetter et al. (2012, 2014, 2017) and Pruszynski et al. (2014) focused on the development of nanobodies, *Camelidiae* derived antibody fragments that are stable, small, and exceptionally specific for their target, HER2. The researchers have labeled these nanobodies with various radionuclides, including ^125^I, ^131^I, or ^177^Lu. It was shown that ^131^I-labeled nanobodies have potential as theranostics by HER2-specific cancer cell binding and internalization, resulting in a significant extension of the median survival in BT474/M1 tumor xenografted mice (D’Huyvetter et al., 2017).

### Mitochondria

Although the current paradigm in radiobiology posits that nuclear DNA is the primary target for ionizing radiation, recent studies provide evidence that extranuclear radiation can have detrimental effects on cell viability as well. The mitochondria have emerged as an interesting but relatively understudied extranuclear target. Circular mitochondrial DNA, like genomic DNA, is sensitive to the ionizing radiation-induced damage. Besides this, some investigators have suggested that ionizing radiation can alter mitochondrial function, induce mitochondrial oxidative stress, and cause mitochondrial-induced apoptosis (Kam and Banati, 2013). An elegant study by Yu et al. (2018) demonstrated this concept with mitochondria-targeting ^177^Lu-porphyrin-PEG nanocomplexes. It was shown that these nanoconstructs, containing the radionuclide ^177^Lu and a photosensitizer, caused an increase in ROS and a reduction in cell viability, in particular when combined with photodynamic therapy. The treatment with and without photodynamic therapy also resulted in a significant tumor growth reduction. Other researchers, such as Maucksch et al. (2016), found indirect evidence of such an effect. They compared the radiotoxicity of three ^99m^Tc-labeled pharmacons with differences in subcellular distribution and found that clonogenic survival was not exclusively determined by the DNA DSB response. They therefore speculated that the observed difference in clonogenic survival of three vectors of ^99m^Tc was the result of a differential mitochondrial accumulation.

### Cell Membrane

Ionizing radiation has detrimental effects on the cell membrane. Hydroxyl radical molecules that are formed as a result of irradiation can attack polyunsaturated fatty acid residues of phospholipids that constitute the cell membrane, leading to the formation of mutagenic malondialdehyde and 4-hydroxynonenal. Furthermore, ionizing radiation is known to activate acid sphingomyelinase, which hydrolyses cell membrane sphingomyelin to produce phosphoryl choline and ceramide. Ceramide is a second messenger for cell apoptosis, and essential for the formation of lipid rafts, which are ceramide-enriched platforms that contain signaling and transport proteins involved in MAPK signaling and sustained ROS and reactive nitrogen species (RNS) production (Pouget et al., 2015). The Pouget group has published several papers in which they have explored the concept of radionuclide-induced cell membrane damage (Pouget et al., 2008; Santoro et al., 2009; Paillas et al., 2016). In one study, a non-internalizing ^125^I-labeled monoclonal antibody against carcinoembryonic antigen (CEA) was compared to an internalizing antibody against EGFR. It was found that internalization is not a prerequisite for effective treatment with AE-emitters *in vitro* and *in vivo*, indicating non-DNA targeting effects (Pouget et al., 2008; Santoro et al., 2009). Paillas et al. (2016) extended this work and demonstrated that the efficacy of the ^125^I-labeled antibody was associated with various factors involved in or affected by the stability of lipid rafts.

### Endosomes and Lysosomes

The targeting of receptor-mediated endocytosis with vectors directed against cell membrane receptors is a strategy that is often used in TRT. Binding of an agonist to its receptor can lead to clathrin-mediated internalization via the formation of plasma membrane vesicles. These vesicles typically fuse into early endosomes, and are subsequently sorted to be recycled, degraded via lysosomes, or modified more specifically in the trans Golgi network (TGN; Scott et al., 2014; Kaksonen and Roux, 2018). Internalization of the receptor–agonist complex provides an opportunity for protracted irradiation of the cell due to retention, as well as the advantage of being closer to sensitive organelles such as the nucleus and mitochondria. The construct lifespan in the endocytic pathway can vary between vectors. For instance, EGF-EGFR complexes are degraded within 5 h, whereas certain nanoparticulate formulations can take days (Song et al., 2015; Dutta et al., 2016). These differences impact the efficacy of the treatment and should therefore be taken into consideration when selecting radionuclides with suitable half-lives.

Two extensively researched TRT targets, PSMA and the somatostatin receptor, exploit this concept. PSMA is a transmembrane glycoprotein that is frequently overexpressed in prostate cancer. Various radiolabeled PSMA-binding peptides and antibodies have been developed for diagnosis and treatment and have been shown to internalize upon receptor association. Targeting PSMA with ^177^Lu-labeled ligands has been particularly successful in the clinic (Eiber et al., 2017), as it is associated with relatively few side effects, a high frequency of objective tumor responses, and a decline in prostate specific antigen (PSA) level (Rahbar et al., 2017). More recently, it has been shown that treatment with ^225^Ac-labeled PSMA-binding peptide resulted in tumor control in a cohort of 40 patients (Kratochwil et al., 2018). Interestingly, a study by Zhu et al. (2016) demonstrated that the internalization pattern of ^225^Ac-PSMA could be changed by conjugation of PSMA ligand to nanovesicles compared to the PSMA ligand alone. It was found that the nano-conjugated PMSA ligand localized in the perinuclear region, whereas PSMA ligand itself remained close to the cell membrane. This perinuclear accumulation translated into a threefold higher cytotoxicity for a given amount of internalized radioactivity, indicating the relevance of subcellular targeting with α-emitters. Other notable examples include the use of ^211^At- and ^125^I-PSMA ligand conjugates, which have shown tumor growth inhibition *in vivo* (Kiess et al., 2015, 2016).

The somatostatin receptor family is upregulated in neuroendocrine tumors and has been evaluated as a target for TRT for over 25 years. As for PMSA, the receptor–ligand complex internalizes upon binding, allowing the payload to irradiate from inside vesicles and lysosomes. The best characterized compound is ^177^Lu-DOTATATE, which has recently shown an impressive increase in progression-free survival and at 20 months in patients with mid-gut neuroendocrine tumors in a phase III clinical trial (65.2% in the ^177^Lu-DOTATATE group versus 10.8% in the control group; Strosberg et al., 2017).

Although DNA is viewed as the primary target for the radiotoxic effects of TRT that bind surface receptor ligands, their internalization can have effects on other structures, including components of the endocytic pathway. Lysosomes are membrane-bound round-spherical vesicles containing hydrolytic enzymes that break down a variety of molecules. As for the cell membrane, the phospholipid layer separating the lysosome content from the cytosol is sensitive to attacks from reactive hydroxyl radicals. Since the majority of intracellular redox-active iron resides in lysosomes, H_2_O_2_ formation may result in labile lysosomes that release lytic enzymes and low mass iron, which can contribute to apoptotic/necrotic death upon prolonged exposure (Persson et al., 2005).

## Concluding Remarks

A discussion about subcellular targeting for therapeutic advantage would not be complete without a consideration of the potential risks associated with unintentional and non-specific accumulation in normal tissue (Howell, 2011). It is well documented that the risks associated with low dose and low dose rate exposures encountered in diagnostic nuclear medicine are minimal (ICRP, 2007). However, the inherent risks associated with therapeutic nuclear medicine procedures are inevitably higher. In this regard, the toxicity of a given radiopharmaceutical has to be considered on an individual basis taking into account the dose, dose rate, radiation spectra, and subcellular distribution. It is evident that the highly non-uniform distribution of radionuclides among cell populations has a profound impact on the associated toxicity of a given radiopharmaceutical and that this effect applies to normal as well as malignant tissue.

In the past 10 years, the clinical value of TRT has been demonstrated for the treatment of various cancer indications, resulting in a 38% increase in their use in the United Kingdom between 2007 and 2012 (Rojas et al., 2015; Gill et al., 2017). Given the positive clinical trial results for ^177^Lu-DOTATATE and ^177^Lu-PMSA ligands, the use of TRT in cancer medicine is likely to expand. This should support development of the clinical expertise and infrastructure needed for adoption of new agents into clinical practice. Using radionuclides that are specifically targeted to subcellular structures can greatly improve the efficacy and safety of TRT, and may therefore be an attractive avenue to explore. Many cancer-specific targets reside intracellularly and opening the potential to target these with TRT is predicted to provide a welcome opportunity for the treatment and imaging of cancer.

In this review, we have described the importance of adequate subcellular targeting, and how novel radiopharmaceuticals can be characterized according to their distribution in subcellular compartments. In reality, many drugs will home to several different organelles, complicating the analysis of which are the critical targets. The development of novel subcellular TRT goes hand-in-hand with improvements in techniques to image and determine their exact cellular localization and mechanism of action. The techniques highlighted here provide a good indication of the variation in radionuclide distribution but lack the option to image and measure the localization and effects in live cells with high spatial resolution. Efforts aimed to address this could have far reaching effects for the maturation of the field of targeted subcellular radionuclide therapy.

## Author Contributions

BB, BL, MG, NF, and KV contributed to the ideas and structure of the paper. BB and MG wrote the introduction. BB, BL, and NF developed the radiobiology and methods of detection section. BB and MG wrote the subcellular targets for radionuclide therapy section. BB and NF wrote the conclusion. BL and NF performed the simulations. KV supervised the project, developed ideas, and edited the final manuscript. All authors discussed the paper and contributed to the final manuscript.

## Conflict of Interest Statement

The authors declare that the research was conducted in the absence of any commercial or financial relationships that could be construed as a potential conflict of interest.
